# Zelda Binding in the Early *Drosophila melanogaster* Embryo Marks Regions Subsequently Activated at the Maternal-to-Zygotic Transition

**DOI:** 10.1371/journal.pgen.1002266

**Published:** 2011-10-20

**Authors:** Melissa M. Harrison, Xiao-Yong Li, Tommy Kaplan, Michael R. Botchan, Michael B. Eisen

**Affiliations:** 1 Department of Molecular and Cell Biology, University of California Berkeley, Berkeley, California, United States of America; 2 Howard Hughes Medical Institute, University of California Berkeley, Berkeley, California, United States of America; 3 The California Institute for Quantitative Biosciences, University of California Berkeley, Berkeley, California, United States of America; The University of North Carolina at Chapel Hill, United States of America

## Abstract

The earliest stages of development in most metazoans are driven by maternally deposited proteins and mRNAs, with widespread transcriptional activation of the zygotic genome occurring hours after fertilization, at a period known as the maternal-to-zygotic transition (MZT). In *Drosophila*, the MZT is preceded by the transcription of a small number of genes that initiate sex determination, patterning, and other early developmental processes; and the zinc-finger protein Zelda (ZLD) plays a key role in their transcriptional activation. To better understand the mechanisms of ZLD activation and the range of its targets, we used chromatin immunoprecipitation coupled with high-throughput sequencing (ChIP-Seq) to map regions bound by ZLD before (mitotic cycle 8), during (mitotic cycle 13), and after (late mitotic cycle 14) the MZT. Although only a handful of genes are transcribed prior to mitotic cycle 10, we identified thousands of regions bound by ZLD in cycle 8 embryos, most of which remain bound through mitotic cycle 14. As expected, early ZLD-bound regions include the promoters and enhancers of genes transcribed at this early stage. However, we also observed ZLD bound at cycle 8 to the promoters of roughly a thousand genes whose first transcription does not occur until the MZT and to virtually all of the thousands of known and presumed enhancers bound at cycle 14 by transcription factors that regulate patterned gene activation during the MZT. The association between early ZLD binding and MZT activity is so strong that ZLD binding alone can be used to identify active promoters and regulatory sequences with high specificity and selectivity. This strong early association of ZLD with regions not active until the MZT suggests that ZLD is not only required for the earliest wave of transcription but also plays a major role in activating the genome at the MZT.

## Author Summary

The newly fertilized eggs of most animal species begin development with a series of rapid cell divisions. During this time of rapid DNA replication, there is little or no transcription of the embryo's genome, with the synthesis of new proteins being directed by a store of maternally deposited mRNAs. Several hours after fertilization, at a period known as the maternal-to-zygotic transition (MZT), transcription of the embryo's genome begins in earnest, but little is known about how this process is initiated. In this paper we investigate the role of a protein known as Zelda (or ZLD) at the MZT in the laboratory model insect *Drosophila melanogaster*. ZLD had been previously shown to control the activation of a small number of genes expressed prior to the MZT. Here, using an experimental technique (ChIP-Seq) that allowed us to visualize where on the genome a protein is bound, we show that, approximately an hour prior to the MZT, ZLD is bound to most of the genomic regions active at the MZT. This suggests that ZLD may act as a kind of an “on switch” for the zygotic genome, poising regions where it binds for activation at the MZT, and this raises the possibility that similar master regulators of the MZT exist in other species.

## Introduction

Delayed activation of the zygotic genome during the early phases of embryogenesis is a nearly universal phenomenon in metazoans. Immediately following egg activation, the zygotic genome is largely transcriptionally quiescent, with development controlled by maternally contributed mRNAs and proteins [1], [2]. At a point known as the maternal-to-zygotic transition (MZT) the degradation of maternally provided RNAs is tightly coordinated with widespread initiation of zygotic transcription. Despite the ubiquity of these events, we are only beginning to understand how the zygotic genome is activated at this discrete developmental timepoint.

In *Drosophila melanogaster*, the fertilized egg undergoes a series of replication cycles without cytoplasmic divisions to generate a syncytial blastoderm [3]. During cycle 14, the blastoderm nuclei cellularize and general zygotic transcription is initiated [3]–[5]. However, a subset of genes required for sex determination, pattern formation and cellularization are transcribed as early as cycle 8 [6]. These genes share a common set of related heptameric DNA motifs, CAGGTAG and related “TAGteam” elements, in their regulatory regions, the removal of which abolishes early activation [7].

Several factors present in the early embryo that bind to TAGteam elements have been identified [8]–[10], but accumulated evidence suggests that the zinc-finger transcription factor Zelda (ZLD) is the most important in regulating early gene expression. Mutations in *zld* lead to defects in early embryonic mitosis and severe cellularization defects by mitotic cycle 14 [10], [11]. A microarray study of ZLD-depleted embryos identified 120 genes whose proper expression during early embryogenesis is dependent on ZLD [10], but the full range of ZLD targets and its mechanisms of action are not known.

We have, for several years, been investigating the genome-wide binding of the transcription factors that regulate the anterior-posterior and dorsal-ventral patterning of transcription during and immediately following the MZT. We used chromatin immunoprecipitation coupled with DNA microarray hybridization (ChIP-chip) to identify the regions bound during mitotic cycle 14 by 21 of these patterning transcription factors. While the regions bound by any particular factor are, predictably, enriched for its target sequence, in virtually every case the most strongly enriched sequence was not the specific target, but CAGGTAG [12]. This striking and unexpected observation suggested that, in addition to its established role in regulating early transcriptional activation, ZLD might play a central role in regulating genome activity at the MZT. Here we investigate this possibility using chromatin immunoprecipitation coupled with high-throughput sequencing (ChIP-Seq) to determine the genomic landscape of ZLD binding as the embryo progresses through the MZT.

## Results

### ChIP-Seq on individually staged and hand-sorted embryos

Although we were particularly interested in the possible role of ZLD at the MZT (mitotic cycle 14), we felt it was essential that we investigate ZLD binding when it is known to activate early zygotic transcription, as well as at the onset of and during the MZT. We therefore collected embryos from population cages and fixed them for chromatin extraction at three timepoints following egg-laying: 60–90 minutes, targeting mitotic cycles 8 and 9 when ZLD levels increase [9], [11] and the earliest zygotic transcription occurs [6]; 120–150 minutes targeting mitotic cycle 13 and early mitotic cycle 14 when widespread zygotic transcription begins; and 180–210 minutes targeting late mitotic cycle 14 when robust zygotic transcription has been established.

In a typical ChIP experiment, chromatin would be prepared directly from these timed embryo collections. However, *D. melanogaster* females do not always lay eggs immediately following fertilization, meaning that while these bulk embryo collections were timed to target a particular stage, they invariably contained a small number of older embryos. Since, at this stage of development, even moderately older embryos contain substantially more DNA, even a small fraction of contaminating older embryos can represent a substantial fraction of purified chromatin. We therefore hand sorted each pool by individually examining every embryo under a light microscope and removing those that did not have the distinguishing morphological characteristics of the stage that sample was targeting.

Through this laborious procedure we obtained pure pools containing approximately 1, 0.2, and 0.1 g of embryos respectively for cycles 8–9, late cycle 13 and early cycle 14, and late cycle 14 (Figure S1). For simplicity, in the rest of the manuscript, we will refer to these samples as cycle 8, cycle 13 and late cycle 14 respectively, although we want to emphasize that we sorted embryos based on morphology and not directly on mitotic cycle, and each sample contained a mix of embryos at adjacent mitotic cycles.

We performed immunoprecipitations using previously described affinity-purified anti-ZLD antibodies [9]. To avoid possible cross reactivity between these antibodies and other zinc-finger containing transcription factors, we depleted our antibody pool of antibodies that recognize any of the four zinc fingers that comprise the DNA-binding domain. We sequenced immunoprecipitated DNA on an Illumina Genome Analyzer IIx, mapped reads to the *D. melanogaster* reference sequence using Bowtie [13], and identified peaks using the Grizzly Peak-finding algorithm (see Materials and Methods). These data represent the first genome-wide analysis of ZLD binding, and, to our knowledge, the first genome-wide analysis of transcription factor binding as the embryo proceeds through zygotic genome activation at the MZT.

### ZLD is bound to thousands of sites prior to the MZT

**Figure 1:**
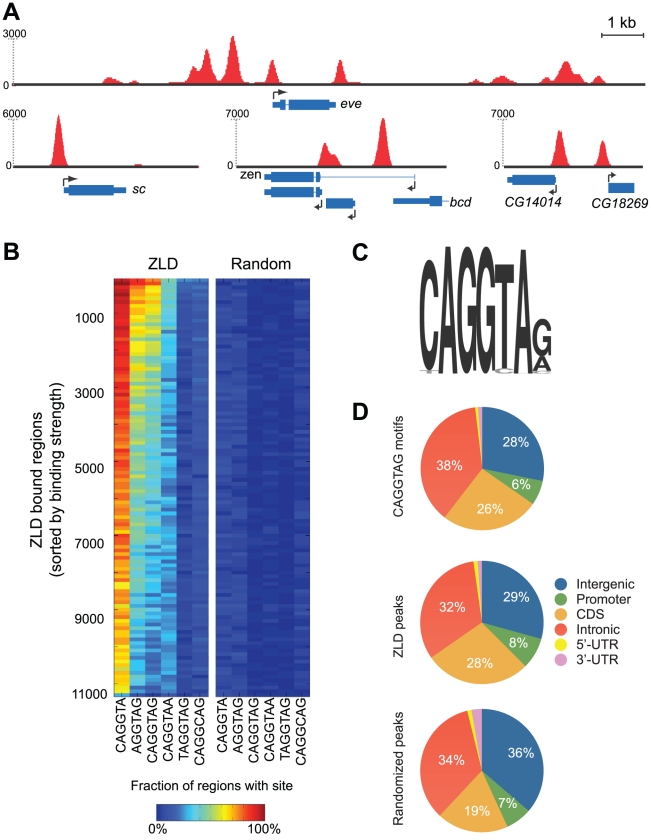
ZLD binds to TAGteam elements in promoters and regulatory elements prior to zygotic activation. (A) Snapshots of ZLD binding at cycle 8 in the loci containing the early-transcribed genes *scute* (*sc*), *even-skipped* (*eve*), *zerknüllt* (*zen*), CG14014 and CG18269 (B) Enrichment of ZLD binding site variants among ZLD peaks from cycle 8. Peaks were sorted from highest (top) to lowest (bottom) levels of binding and binned into groups of 100. The percent of peaks in each bin containing the appropriate motif within 150 bp of the peak location is indicated by the color of the cell in the left panel, the percent of peaks containing the motif after random repositioning of peaks is shown in the right panel. (C) Sequence motif identified from top 500 ZLD peaks using MEME [14] (D) Distribution of genomic annotations among occurrences of CAGGTAG (top), ZLD peaks at cycle 8 (center), and the expected distribution of annotations (based on their genomic abundance) after randomly repositioning the peaks across the genome (bottom).

Although we used a relatively small amount of input chromatin for each sample, the ChIP-Seq data were of high quality, with well-resolved peaks and high signal-to-noise ratio (Figure 1A). We identified 11,374 peaks at cycle 8, 10,471 peaks at cycle 13, and 9,432 peaks at late cycle 14 (Tables S1, S2, S3).

We analyzed these regions for enriched occurrences of sequence motifs using a variety of computational algorithms [14]–[16], and consistently recovered the previously identified 7-mer CAGGTAG [7], [9], [10] and several variants as the primary determinants of ZLD binding in vivo (Figure 1B and 1C). For example, 66% of the top 1,000 ZLD peaks at cycle 8 contain the CAGGTAG motif at least once, as opposed to the random expectation of 1.8%. Similarly, CAGGTA and AGGTAG, two shorter versions of the motif, appear, respectively, in 94% and 75% of the most highly bound regions. There is also a strong correlation between the number of occurrences of CAGGTAG in a region and the magnitude of ZLD binding (Figure S2). Surprisingly, other TAGteam elements, including TAGGTAG and CAGGCAG, which were shown by mutation analysis to participate in the early expression of *scute* (*sc*) [7], and to be bound by ZLD in vitro [9], [10], were not significantly enriched among the top 1,000 regions bound in vivo.

As previous experiments implicated ZLD in the activation of early zygotic expression [10], [11], we focused our attention on binding at cycle 8, when zygotic transcription is initiated. We observed strong ZLD binding to many genes in cycle 8 embryos, including the early-transcribed genes *sc*, *zerknüllt* (*zen*) and *even-skipped* (*eve*) (Figure 1A). ZLD was found at the promoters of 1,171 genes at cycle 8. However, promoters (defined here as 500 bp upstream to 150 bp downstream of the transcription start site) represented only eight percent of ZLD-bound regions, with the remainder distributed evenly across gene bodies and non-coding DNA (Figure 1D). The observed distribution of bound regions closely mirrors the distribution of the CAGGTAG motif across the genome (Figure 1D). Indeed, we find that 64% of CAGGTAG sites are bound by ZLD in cycle 8 embryos, indicating that ZLD's inherent affinity for DNA, rather than interactions with other factors or chromatin structure, is the major determinant of its binding at this early stage.

### ZLD directly regulates transcriptional activation in the early embryo

**Figure 2:**
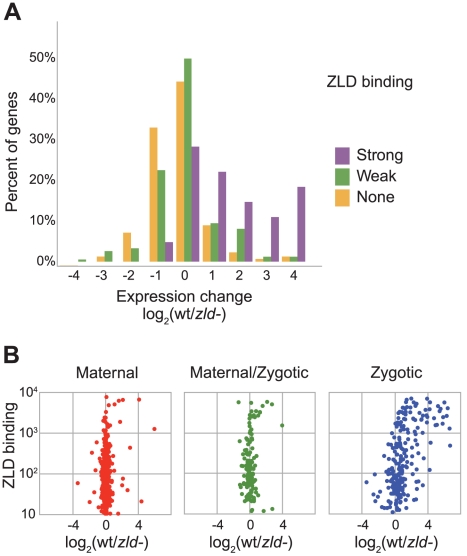
Zygotically transcribed ZLD targets are downregulated in embryos depleted for maternal ZLD. (A) Gene expression differences between wildtype embryos and ZLD-depleted embryos [10], among 101 zygotic genes with strong (greater than 500) ZLD cycle 8 binding at promoters (purple), at 270 zygotic genes with weaker ZLD promoter binding (green), and among 1639 zygotic genes with no ZLD promoter binding (orange). Zygotic genes are as defined in [17] (B) Comparison of effect of ZLD depletion and ZLD promoter binding at cycle 8 among maternally deposited genes with no zygotic expression in early embryo (left), genes that are both maternally deposited and zygotically transcribed (center), and genes with exclusively zygotic transcription (right). Classification of genes is as defined in [17].

In their paper describing ZLD as a CAGGTAG binding protein, Liang et al. [10] used microarrays to measure expression differences between wildtype cycle 8–13 embryos and those lacking maternal ZLD. Although they identified 120 genes down-regulated in ZLD-depleted embryos, they could not determine how much of this effect was directly due to the actions of ZLD. To see if we could resolve this ambiguity, we compared their genome-wide mutant expression data to our ZLD binding data, and found a very strong association between ZLD binding and expression. In particular, most genes strongly bound by ZLD at their promoters during cycle 8, and detectably expressed in cycle 8–13 wildtype embryos, were downregulated in embryos lacking ZLD (Figure 2A). The effect is more pronounced when we exclude maternally deposited mRNAs (using data from [17]), as the expression effect of ZLD binding is restricted to zygotically transcribed genes (Figure 2B). These analyses suggest that the expression effects observed by Liang et al. were largely direct, and that ZLD binding to promoters is required for zygotic activation of the small number of genes transcribed in the early embryo.

### ZLD binds early to the promoters of genes subsequently transcribed at the MZT

**Figure 3:**
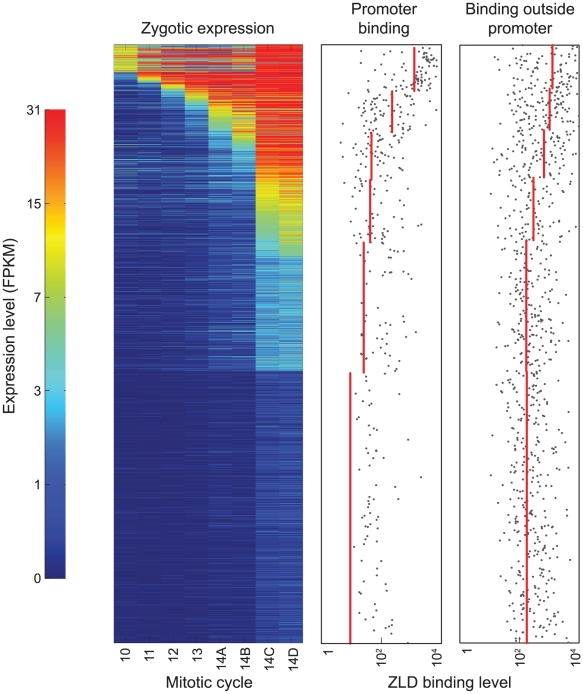
At mitotic cycle 8, ZLD marks promoter and non-promoter regions of genes activated at the MZT. The left panel shows the expression patterns of 2,010 genes with exclusively zygotic expression at eight timepoints before and during the MZT (list of zygotically transcribed genes and expression data from [17]). Genes are sorted from early (top) to late (bottom) onset of zygotic transcription. The two panels on the right show the levels of ZLD binding to promoter and non-promoter regions for each gene (grey dots; regions with no detected binding are not plotted). Red lines show average levels of ZLD binding in groups of genes with similar times of transcription onset.

Given this strong relationship between ZLD promoter binding in cycle 8 embryos and changes in transcription upon ZLD depletion, we next examined the relationship between ZLD binding and the onset of zygotic transcription in wildtype embryos. We took advantage of a recently published high-resolution time course of zygotic gene expression in the early embryo [17], and compared ZLD binding at the promoters of 2,010 genes with exclusively zygotic expression to the time at which the genes are first detectably transcribed (Figure 3).

Surprisingly, the promoters of many genes that are not expressed until cycle 14 were already bound by ZLD at cycle 8. For example, the genes *odd-paired* (*opa*) and *leak* (*lea*) are not expressed until mitotic cycle 14, but were highly bound by ZLD at cycle 8 (Figure S3). More generally, there was a strong correlation between the strength of cycle 8 ZLD promoter binding and the onset and magnitude of gene expression (Figure 3), with higher levels of cycle 8 promoter binding associated with earlier and stronger expression.

### Early ZLD binding marks sites later bound by zygotically expressed transcription factors

While ZLD binding at promoters is strongly associated with zygotic transcription, the widespread binding of ZLD to non-promoter regions of genes active in the early embryo suggests a more general role in activating the zygotic genome. As shown in Figure 1D, more than 90 percent of the regions bound by ZLD are outside of promoter regions. And, as with promoter binding, there is a high correlation between ZLD binding in non-promoter regions and the timing and magnitude of zygotic expression (Figure 3).

**Figure 4:**
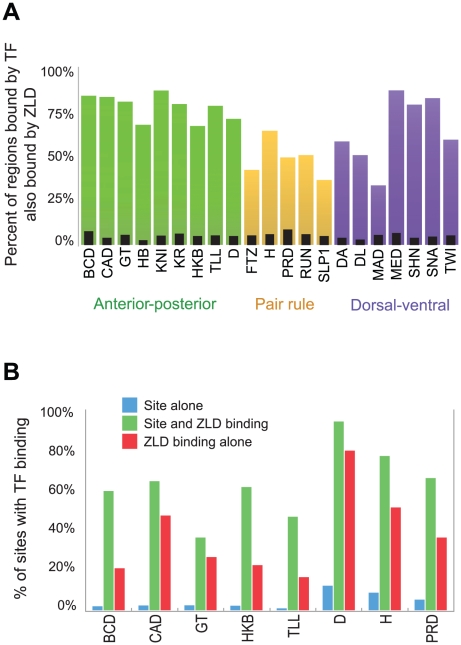
ZLD binding precedes and overlaps transcription-factor binding in regulatory sequences. (A) Fraction of regions highly bound (top 300 regions from [18]) by each of 21 transcription factors involved in anterior-posterior or dorsal-ventral patterning at cycle 14 that are bound by ZLD at cycle 8. As a control, narrow black bars show coverage after random reshuffling of ZLD-bound regions (B) For eight factors from [18], we compare the fraction of genome-wide recognition site occurrences that are occupied by each factor (blue), to the fraction of occupied sites that occur in regions that were bound by ZLD in cycle 8 (green). The significant increase in the probability of a factor binding to its own recognition sites in ZLD-bound regions emphasizes the role that ZLD plays in activation of the zygotic genome. Red bars mark the expected percent of TF-occupied sites among ZLD peaks, even in the absence of a TF-recognition element. We analyzed only the eight factors with clear simple recognition elements based on in vitro binding data.

As discussed in the introduction, many regions bound by the transcription factors that establish anterior-posterior (A-P) and dorsal-ventral (D-V) patterning in the early embryo are significantly enriched for CAGGTAG and other ZLD binding sites [12], [18]. We therefore compared ZLD binding at cycle 8 to genome-wide binding measurements of 21 transcription factors involved in A-P and D-V patterning [12], [18]. A strikingly large fraction of the regions most strongly bound by these factors in the cellular blastoderm at mitotic cycle 14 (which several lines of evidence suggest are functional enhancers [12], [18]) are already bound by ZLD at cycle 8 (Figure 4A). Given that only four of these factors (BCD, CAD, GT and KR) are present in the embryo at cycle 8, ZLD must be bound to this large collection of enhancers prior to the binding of most of these additional transcription factors—at least four nuclear divisions prior in the majority of cases.

To examine whether ZLD binding affects subsequent transcription factor binding or is simply associated with it, we examined the relationship between the presence of transcription factor target sequences, ZLD binding and transcription factor binding for the subset of factors whose binding specificity is known. As expected, the presence of a target sequence alone is a poor predictor of binding of the corresponding factor (Figure 4B, blue bars), presumably because many of these sequences are found in regions of closed chromatin [19]. However, when we restrict this analysis to regions bound early by ZLD the predictive power of these motifs increases dramatically (Figure 4B, green bars), suggesting that ZLD binding plays a significant role in determining which regions of the genome are accessible to transcription factor binding.

**Figure 5:**
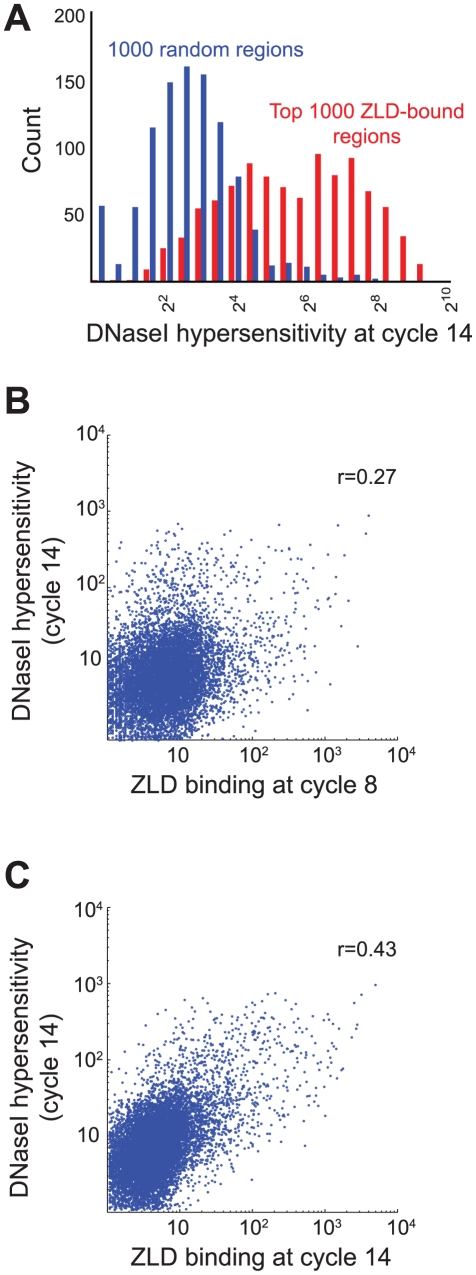
Early ZLD binding predicts chromatin state at the MZT. (A) Histogram of DNA accessibility based on DNaseI hypersensitivity from [20] among the most strongly bound 1,000 ZLD peaks at cycle 8 (red) and among randomly shuffled peak positions (blue). (B) Comparison of genome-wide ZLD binding at cycle 8 (x-axis) vs. the DNaseI hypersensitivity values at cycle 14 [20], showing a genome-wide correlation coefficient of 0.27 (C) Same as (B), but using ZLD binding from late cycle 14, showing correlation coefficient of 0.43.

We next directly examined the relationship between ZLD binding and chromatin accessibility, using recently published DNAseI accessibility from cycle 14 embryos [20]. We found that ZLD binding at cycle 8 was strongly predictive of DNAseI accessibility at cycle 14, with regions bound strongly by ZLD at cycle 8 highly enriched for regions of open chromatin at cycle 14 (Figure 5A). There is also a strong correlation between the amount of ZLD binding at cycle 8 and regions of high DNA accessibility at cycle 14 (Figure 5B; r = 0.27). The relationship between ZLD binding at late cycle 14 and DNAse accessibility at cycle 14 was even stronger (Figure 5C; r = 0.43).

**Figure 6:**
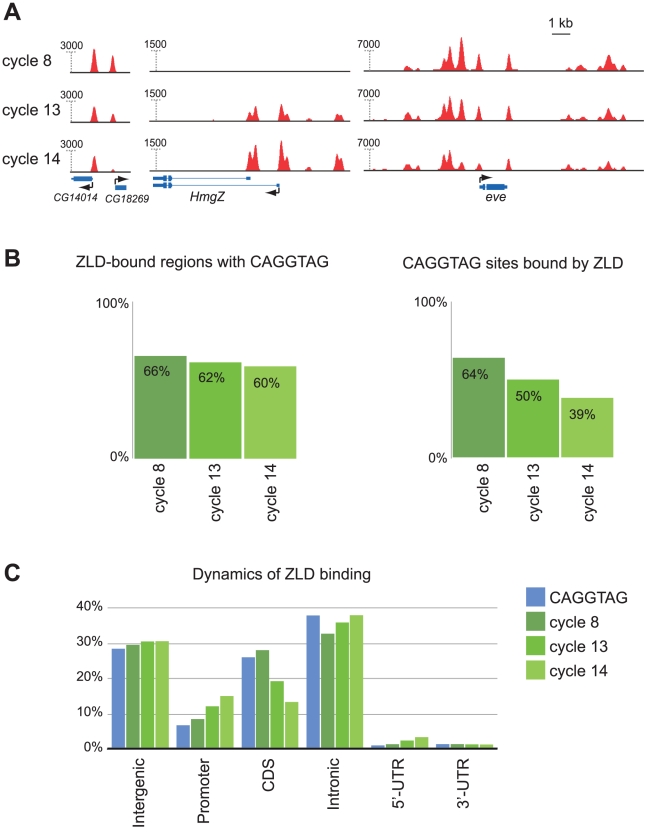
ZLD binding dynamics through the MZT. (A) Dynamics of ZLD binding during cycle 8 (top track for each gene), cycle 13 (middle track), and late cycle 14 (bottom track) at three genomic loci: and CG14014/CG18269, *HmgZ*, and *even-skipped* (*eve*) (B) Specificity and sensitivity of the relationship between CAGGTAG and ZLD binding at the three timepoints. Shown are the fractions of the top 1,000 ZLD peaks that contain the motif at the three timepoints (left), and the fraction motif occurrences that are bound by ZLD (right). (C) Distribution of genomic annotations among genomic occurrences of the CAGGTAG motif (blue) and ZLD peaks from three timepoints (green).

The increasing conformity of ZLD binding to chromatin state piqued our interest in the dynamics of individual ZLD binding sites. ZLD binding is fairly stable over time: of 12,135 peaks found in pooled data from the three stages, 10,873 (90%) are found in all three stages (Figure S1B and Table S4). For example, ZLD is bound at all three stages to genes such as *sc* and *eve* that are transcribed prior to the MZT, as well as to genes such as *lea* and *opa* that are expressed only later. There are, however, clear changes in binding. For example, 775 sites are present in cycle 8 embryos but absent at late cycle 14 (Figure 6A, Figure S1B, and Table S4). This dynamic binding is specific to individual bound regions, as we identified many loci where ZLD binding at one site remained unchanged while binding to a neighboring binding site increased or decreased.

An interesting pattern emerged when we examined the relationship between ZLD binding, TAGteam sites and gene annotations at the three developmental stages. At cycle 8 there is a very strong relationship between occurrences of CAGGTAG and ZLD binding (Figure 6B): over 66% of the top 1,000 ZLD bound regions contain a CAGGTAG site (compared to 1.8% expected at random), while an astonishing (relative to other studied factors) 64% of genomic CAGGTAG sites are indeed bound by ZLD. The unusually high fraction of CAGGTAG sites bound by ZLD at cycle 8 suggests that chromatin at this stage is in a fairly accessible state. By cycle 13, only 50% of CAGGTAG sites are bound (Figure 6B), as ZLD binding becomes more enriched in promoter sequences and less enriched among coding regions (Figure 6C). And by late cycle 14, only 38.5% of the CAGGTAG sites are bound (Figure 6B) and the shift from coding region to promoter binding continues (Figure 6C). The decreasingly specificity of ZLD for CAGGTAG sites over time suggests that the chromatin landscape is becoming more differentiated, and may also reflect the larger number and greater diversity of DNA-binding proteins present after zygotic transcription begins.

## Discussion

### A model for ZLD as a pioneer transcription factor that shapes the chromatin landscape at the MZT

ZLD and the TAGteam sequences to which it binds were originally identified as key regulators of the early wave of zygotic transcription that precedes the MZT [7], [10], and our genome-wide measurements of ZLD binding validate this activity. However, we have demonstrated that ZLD is also bound to the promoters and enhancers of more than a thousand genes that are not transcribed until the MZT, and that early ZLD binding is strongly associated with open chromatin and transcription factor binding during the MZT. Thus, rather than being specifically involved in the onset of zygotic transcription, our data indicate that ZLD has a much wider role in activating the zygotic genome, although its specific molecular mechanism remains elusive.

The sequence of ZLD offers few clues to its function. Its roughly 1,600 amino acids contain no known domains besides C2H2 zinc-fingers, and none of its orthologs (found only in arthropods) have been experimentally characterized [10], [11], [21]. That ZLD is important in both promoters and enhancers, and that its binding seems to affect the distribution of a diverse collection of transcription factors, argue against it directly recruiting polymerase and transcription factors. We propose instead that ZLD acts as a generic activator of the zygotic genome by controlling chromatin accessibility and/or histone modifications in the regions where it is bound.

There is increasingly good evidence that difference in chromatin state across the genome at the MZT play a major role in determining which regions are active. We and others have recently assayed the state of chromatin in cycle 14 embryos and shown that regions of concentrated transcription factor binding are strongly associated with regions of “open” chromatin [22], and that temporal changes in DNA accessibility and transcription factor binding are often coordinated [20]. Furthermore, a recent computational analysis from our lab that dissected the factors that influence our ability to predict transcription factor binding offers compelling evidence that, at least in the *D. melanogaster* blastoderm, the state of chromatin shapes—and does not simply reflect—transcription factor binding [19]. But one important question left unanswered by these studies is how differences in chromatin state are established. Our data and analyses clearly implicate CAGGTAG sites and ZLD.

We already knew that CAGGTAG sites were enriched in active promoters and regions of transcription factor binding at the MZT [7], [10], [12], [18], and that the gain and loss of CAGGTAG sites is a major driver of changes in transcription factor binding at the MZT between different *Drosophila* species [23]. Here we have shown that ZLD binds to these CAGGTAG sites in vivo; that there is a tight connection between ZLD binding, chromatin state and MZT activity; and, crucially, that ZLD binding precedes, by at least several mitotic cycles, transcription factor binding and transcription at regions active at the MZT. Thus it is in precisely the right places at the right time to act as a generic activator of the MZT.

Although little is known about the chromatin state in the early embryo, our data support a model in which the genome transitions from a fairly uniform open state (in which ZLD binds to 65% of CAGGTAG sites) to the mosaic of open and closed domains known to exist in cycle 14 [22] (and in which ZLD binds to only 39% of CAGGTAG sites). If this is correct, we suggest ZLD likely plays a role in managing this transition, recruiting or repelling chromatin remodeling proteins to the regions where it is bound in uniformly open chromatin at cycle 8 and thereby ensuring they remain open at cycle 14. It is, however, also possible that early ZLD binding to its MZT targets may represent opportunistic binding of the protein to accessible regions containing CAGGTAG sites, with its MZT-specific activity arising from binding closer in time to the MZT.

ZLD shares some compelling similarities with *Xenopus* β-catenin, which is required for expression of a subset of genes prior to the MZT [24]. At least two genes, *siamois* and *xnr3*, require β-catenin for expression, but are not expressed until the MZT. β-catenin is required at or before the 32 cell stage to poise *siamois* and *xnr3* for activation and helps to establish this poised state by recruiting the histone methyltransferase Prmt2 to the promoters of these genes [25]. Thus β-catenin and ZLD are similarly required to drive pre-MZT expression of a subset of genes and also to poise additional genes for activation at the MZT. But unlike the specialized function of β-catenin, our data suggest that ZLD acts globally to activate the zygotic genome.

Our proposed function for ZLD is reminiscent of the so-called “pioneer” transcription factors. This concept was introduced to describe the role of FoxA1 in regulating gene regulation in the developing mammalian liver. In the undifferentiated endoderm, FoxA1 is bound to the enhancer of the hepatocyte-expressed albumin gene (*Alb1*) before *Alb1* is expressed [26]. FoxA1 binding mediates chromatin decondensation, and this modified chromatin environment allows for the subsequent binding of additional transcription factors that drive liver-specific gene expression [27].

However, in contrast to chromatin in multipotent progenitor cells, the chromatin of the totipotent cells of the early embryo are likely to be in a relatively “open” conformation [28]. Thus, ZLD may not actively mediate chromatin decondensation but rather may act to maintain regions of accessible chromatin. There is precedent for chromatin remodeling being involved in the MZT. In mice, the chromatin-remodeling enzyme BRG1 is required for zygotic genome activation [29].

Work in embryonic stem cells and in zebrafish embryos suggests that transcriptional activation at the MZT also involves specific histone modifications. In zebrafish, histones acquire modification patterns reminiscent of pluripotent embryonic stems cells as the embryo progresses through the MZT [30]. Most notably, histone H3 acquires both marks of active transcription, tri-methylation on lysine four (H3K4me3), and of repression, tri-methylation on lysine 27 (H3K27me3). These bivalent histone marks were initially observed in embryonic stem cells and have been shown to poise the genomes of these cells for differentiation [31]–[33].

Such bivalent marks have not been observed in *Drosophila*. However the earliest embryos examined were 4–12 hours old [34], after the embryo has transitioned through the MZT and its cells are no longer fully pluripotent. Recently, it has been shown that in embryonic stem cells, bivalent domains are resolved as cells differentiate [33], raising the possibility that bivalent domains are present in *Drosophila* but no longer evident in the post-gastrulation embryos that have been examined. Perhaps ZLD works by recruiting or otherwise influencing the recruitment of proteins that modify chromatin, or by modifying chromatin itself. However, the fact that no bivalent domains have been observed in *Drosophila* or in *Xenopus* [35] leaves open the possibility that ZLD is acting through a different mechanism. It is imperative that careful genome-wide analysis of histone modifications be performed in *Drosophila* and other species as they transition through the MZT to determine whether the formation of bivalent chromatin domains is a common characteristic of pluripotent cells.

### What differentiates ZLD target genes expressed prior to the MZT from those genes expressed only later?

The genes most highly-bound by ZLD are transcribed by cycle 10. In one case, it has been shown that increased ZLD binding alone can lead to precocious activation [7], and it is possible that high levels of ZLD binding to promoters and proximal enhancers is sufficient to activate expression. However, most ZLD bound regions are not active until cycle 14. The generally lower levels of ZLD binding to these regions may necessitate the presence of other factors (such as patterning transcription factors or STAT92E [36]) not expressed or activated until closer to the MZT. In this way ZLD would act indirectly to keep chromatin open at these regions until these other factors are able to exert their control. Alternatively, ZLD may act to directly recruit a zygotically expressed coactivator to the regulatory regions of genes expressed at the MZT. For example, ZLD could recruit factors, such as P-TEFb, that work to release stalled RNA polymerase II [37] or, similar to β-catenin, recruit chromatin-modifying enzymes [25]. The ability of ZLD to activate transcription could also be modulated by post-translational modifications to the protein itself.

It is worth noting that before zygotic induction *Drosophila* embryos are undergoing rapid rounds of DNA replication and ORC, the replication initiator, does not bind to specific sequences [38], but rather depends upon access to open chromatin [39]. Hence ZLD, with its potential role in shaping the chromatin landscape may also play a key role prior to transcription initiation in allowing for the proper assembly and spacing of pre-replication sites, and CAGGTAG may be a good predictor of origins [40]. As the embryo progresses through the MZT, ORC binding becomes less closely spaced and origin firing becomes less synchronous suggesting that DNA replication reflects a changing chromatin environment.

It is noteworthy that ZLD may activate distinct sets of genes by different mechanisms. TAGteam sites were first defined as sequence elements driving the expression of a small number of genes prior to the MZT [7]. It was therefore assumed that the TAGteam-binding protein, ZLD, might function specifically to activate this subset of genes. However, we have shown that ZLD is marking the genome for widespread transcriptional activation of the zygotic genome at cycle 14. Perhaps, ZLD is able to directly activate the small subset of genes expressed prior to the MZT, but that ZLD-mediated gene activation at the MZT requires additional zygotically expressed cofactors or post-translational modifications.

### Genome poising as a general feature of animal development

Given the ability of transcription factors such as β-catenin, FoxA1, and ZLD to mark genes for subsequent activation, and the recent evidence that chromatin remodeling, histone modifications and RNA polymerase II occupancy prepare developmental genes for later transcription, we suggest that the poising of genomes for subsequent activation is likely to be a common feature of pluripotent cells. Determining the roles of these mechanisms in regulating gene expression at this important developmental timepoint will be crucial to understanding how these cells are poised for differentiation and how subsequent activation can be regulated to drive specific cell fates.

## Materials and Methods

### ZLD antibody purification

As described in Harrison et al. [9], rabbits were immunized with GST fused to amino acids 1117–1487 of ZLD and purified against the same portion of the protein fused to maltose binding protein (MBP). As this portion of ZLD includes the zinc-finger DNA-binding domain, we further purified these antibodies using MBP fused to the four zinc fingers, amino acids 1318–1444. For our experiments, we recovered the antibodies that failed to bind to this MBP fusion protein and confirmed by immunoblot that these antibodies could recognize the full-length ZLD, but not the DNA-binding domain alone.

### Formaldehyde crosslinking of staged and sorted embryos, and chromatin isolation

*D. melanogaster* flies were maintained in large population cages in an incubator set at standard conditions (25°C). Embryos were collected for 30 minutes, and then allowed to develop for 60, 120 or 180 additional minutes before being harvested and fixed with formaldehyde. The fixed embryos were staged and hand sorted in small batches using an inverted microscope (Figure S1A) to remove the small number of older contaminating embryos resulting from egg retention, with the sorting first done at 4× and then confirmed at 10× magnification. Our visual inspection of all of the processed embryos gave us great confidence that we had removed later-stage contaminants, a view bolstered by an assessment of the trends in ZLD binding over the three timepoints. In particular, the presence of regions with no binding at cycle 8 but high levels of binding in later stages (Figure 6A) demonstrated that our cycle 8 binding was not simply later stage contamination, as did the enrichment for cases in which cycle 13 binding was intermediate between cycle 8 and late cycle 14 (Figure S1B).

1, 0.2, and 0.1 g of embryos at the three different stages respectively, were used to prepare chromatin for immunoprecipitation following the CsCl_2_ gradient ultracentrifugation protocol as previously described [12]. With the small amount of embryos in each sample, the ultra-centrifugation was carried out with a SW41 rotor, and the volumes of buffers, detergents, and CsCl2 solutions were adjusted accordingly as detailed in the previous protocol.

### ChIP and sequencing

The chromatin obtained was fragmented to sizes ranging from 100 to 300 bp using a Bioruptor (Diagenode, Inc.) for a total of processing time of 140 min (15 s on, 45 s off), with power setting at “H”. We used 3.7 µg chromatin from cycle 8, 6.6 µg from cycle 13 and 6 µg from cycle 14 in the chromatin immunoprecipitation reaction, using the affinity purified anti-ZLD antibody, following the procedure described previously [12]. The sequencing libraries were prepared from the ChIP and Input DNA samples, and subjected to ultra-high throughput sequencing on a Solexa Genome Analyzer IIx as previously described [23], except that the DNA fragments ranged from 200–350 bp in size.

### Mapping sequencing reads to the genome

Sequenced reads were mapped to the April 2006 assembly of the *D. melanogaster* genome, (UCSC version dm3, BDGP Release 5) using Bowtie [13] using the command-line options ‘-n 2 -l 36 -m 2’, thereby keeping for further analyses only tags that mapped uniquely to the genome with at most two mismatches. Each read was extended to 150 bp based on its orientation, and the total number of reads per timepoint was normalized to 10,000,000.

### Peak calling

We developed a model-based multi-peak algorithm—Grizzly Peak—to accurately identify significant ZLD bound loci across the genome. Grizzly Peak is an iterative model-based peak fitting method, which we modified from Capaldi et al. [41]. In brief, Grizzly Peak estimates the expected shape of a binding event in the ChIP-seq data. The algorithm then iteratively scans the genome and identifies enriched regions with high protein occupancy. These regions are expanded and analyzed, aiming at finding a minimal set β of peaks (each with a genomic position and an occupancy level) optimizing the fit to the measured data. To allow for overlapping peaks, we devised a simple heuristic for considering actions such as adding or removing peaks. Each step is then assigned a score, and steps are taken if a significant improvement in the score is achieved. Once a genomic region has been analyzed and fitted, the optimized set of peaks is recorded, and this genomic region is discarded from future fitting. This process is repeated until no significantly bound loci remain. The Grizzly Peak algorithm is available at http://eisenlab.org/software/grizzly.

### Motif analysis

Identified peaks were expanded to 300 bp around each binding event (peak center), and were analyzed for enriched motifs. We used three de novo motif discovery tools. First we used MEME (version 4.5.0) [14], searching in a zero or one binding site per peak (“zoops”) mode, and allowing for up to 10 motifs, while testing both strands. In addition, we used another motif analysis algorithm using Expectation-Maximization (EM), and assuming at least one binding site per peak [15]. We accompanied our analysis by Weeder (version 1.4.2) [16], an exhaustive enumeration algorithm that tests the enrichment of each motif among the input sequences.

### Genomic annotations

Each called peak was assigned a genomic functional annotation based on FlyBase gene annotations (UCSC, release dm3), including the position of exons and transcript start and end points. According to the position of the peak center position, we categorized each peak into one of six genomic categories: (1) Promoter peaks – from 500 bp upstream to 150 bp downstream to an annotated start site; (2) Coding sequence (CDS) peaks – overlapping any exon; (3) 5′-UTR peaks – overlapping a transcript, but not CDS or promoter; (4) 3′-UTR peaks; (5) Intron peaks; and (6) Intergenic peaks – downstream of genes or more than 500 bp upstream. Each peak was then assigned to the nearest gene.

### Randomization via genomic shuffling of peak positions

To estimate the random expected distribution of ZLD peaks relative to genome annotations, we devised a simple strategy to assign every peak to a new randomized position that maintained the number of peaks, their sizes, their distribution over chromosomes and their relative distances from each other. First we randomly reordered the peaks in each chromosome, practically mixing between strong and weak peaks. Second, we randomly shuffled the linker distances between every pair of adjacent peaks. Finally, we repositioning each peak at a new randomized position and repeated the analyses at hand.

### Zygotic expression—data, class, and estimate onset time

We used single-embryo zygotic expression data from Lott et al. [17], including gene classification to (1) zygotic; (2) zygotic/maternal; and (3) maternal only. These were done according to the zygotic expression patterns of each gene and its genotypic signature. Onset times for zygotic genes were determined as the first time for each gene with zygotic mRNA abundance above 5 RPKM (reads per kilobase per million reads mapped, limited to autosomal chromosomes), after interpolating the eight measured timepoints using a shape-preserving piecewise cubic interpolation (MATLAB R2010a, interp1 function, “pchip” model).

### Analysis of expression in embryos depleted for maternal ZLD

Raw gene expression data from Liang et al. [10] for wildtype embryos and embryos depleted for maternal ZLD were downloaded from GEO (accession GSE11231) and reanalyzed. Up- and down-regulation were estimated by comparing expression levels in wildtype to ZLD-depleted embryos. Probes marked as “absent” in both strains (“noise” vs. “noise”) were discarded from further analyses of these data.

### Overlap with developmental transcription factors

ChIP-chip data for 21 transcription factors during early developmental stages (cycle 14) were obtained from MacArthur et al. [18], at http://bdtnp.lbl.gov/Fly-Net. We applied Grizzly Peak to identify the exact binding position for each factor within the 1% FDR (symmetrical) enriched regions. We then analyzed the co-occurrence of ZLD peaks vs. the top 300 peaks of each factor. As a control, we repeated this analyses after randomly repositioning all the peaks per TF using the random shuffling approach described above. We then compared the coverage of these randomized positions with ZLD, and calculated the percent of recognition elements that are bound in the presence or absence of ZLD. We focused on eight well-studied factors with simple recognition motifs (BCD: TAATCC; CAD: TTTATTG; GT: TTACGTAA; HKB: GGGCGTG; TLL: TTGACTTT; D: CCATTGT; H: CACGCGCC; and PRD: GTCACGC). We identified all genomic occurrences of these motifs, and calculated the fraction of bound motifs (using a 1% FDR threshold from MacArthur et al. [18]). These fractions were then compared to the number of bound motifs given overlapping ZLD binding (ZLD occupancy above 100 RPKM in late cycle 14). Finally, we repeated this analysis with a randomly shuffled set of genomic positions (instead of the real occurrences of recognition motif for each TF) to test the different basal correlations of each factor and ZLD. Merged data with ZLD binding and data from previously published transcription factors are provided in Table S5.

### Data availability

Raw and mapped sequencing reads are available from the National Center for Biotechnology Information's GEO database (http://www.ncbi.nlm.nih.gov/geo/) under accession number GSE30757. A browser with ZLD binding and other related data discussed in the manuscript can be accessed at http://eisenlab.org/data/ZLD.

## Supporting Information

Figure S1 Hand-sorted embryos and evidence of successful sorting. (A) Hand-sorted embryos used for ZLD chromatin-immunoprecipitation during cycle 8 (left), cycle 13 (center), late cycle 14 (right). (B) Classification of ZLD peaks based on relative binding strength at three timepoints. We identified 12,135 peaks in pooled reads from all timepoints, and then used reads from individual timepoints to compute occupancy at each of these sites and classified peaks based on change from cycle 8 to cycle 13, and from cycle 13 to late cycle 14.
(EPS)

Figure S2 Strength of ZLD binding correlates with number of ZLD target sites. Average number of CAGGTAG (top) and CAGGTA (bottom) motifs within 150 bp of ZLD peaks at cycle 8 (peaks grouped in to bins of 100).
(EPS)

Figure S3 Binding and expression of two genes (*opa* and *leak*) bound by ZLD but not expressed until MZT.
(EPS)

Table S1 ZLD peaks at cycle 8.
(XLS)

Table S2 ZLD peaks at cycle 13.
(XLS)

Table S3 ZLD peaks at late cycle 14.
(XLS)

Table S4 ZLD peaks in combined data.
(XLS)

Table S5 Master data table with ZLD binding, transcription factor and polymerase ChIP, and expression data.
(XLS)
